# Promoting Children’s Physical Activity in Low-Income Communities in Colorado: What Are the Barriers and Opportunities?

**DOI:** 10.5888/pcd14.170111

**Published:** 2017-12-14

**Authors:** Daniel M. Finkelstein, Dana M. Petersen, Lisa S. Schottenfeld

**Affiliations:** 1 Mathematica Policy Research, Cambridge, Massachusetts; 2 Mathematica Policy Research, Oakland, California

## Abstract

**Introduction:**

Colorado has the highest rate of adult physical activity in the United States. However, children in Colorado have a lower rate of physical activity relative to other states, and the rate is lowest among children in low-income households. We conducted focus groups, surveys, and interviews with parents, youth, and stakeholders to understand barriers to physical activity among children in low-income households in Colorado and to identify opportunities to increase physical activity.

**Methods:**

From April to July 2016, we recruited participants from 5 communities in Colorado with high rates of poverty, inactivity, and obesity; conducted 20 focus groups with 128 parents and 42 youth; and interviewed 8 stakeholders. All focus group participants completed intake surveys. We analyzed focus group and interviews by using constant comparison.

**Results:**

We identified 12 themes that reflect barriers to children’s physical activity. Within the family context, barriers included parents’ work schedules, lack of interest, and competing commitments. At the community level, barriers included affordability, traffic safety, illicit activity in public spaces, access to high-quality facilities, transportation, neighborhood inequities, program availability, lack of information, and low community engagement. Survey respondents most commonly cited lack of affordable options and traffic safety as barriers. Study participants also identified recommendations for addressing these barriers. Providing subsidized transportation, improving parks and recreation centers, and making better use of existing facilities were all proposed as opportunities to improve children’s physical activity levels.

**Conclusion:**

In this formative study of Colorado families, participants confirmed barriers to physical activity that previous research on low-income communities has documented, and these varied by geographic location. Participants proposed a set of solutions for addressing barriers and endorsed community input as an essential first step for planning community-level health initiatives.

## Introduction

Regular physical activity has important benefits for children’s health and well-being (1). Although Colorado has the highest rate of adult physical activity in the United States (2), children rank lower on physical activity, and this rate is lowest among children in low-income households (3). A foundation-funded effort is under way to increase physical activity among children in low-income families. Although barriers to physical activity in low-income communities are well-documented (4–7), the foundation recognizes Colorado’s unique demographics and geography and the need to invest in strategies that align with community priorities and characteristics. The foundation funded this formative study to understand families’ views about barriers to children’s physical activity in a sample of low-income Colorado communities — and their solutions for addressing these barriers — as an initial step for planning future investments. This type of community-informed approach is essential for identifying strategies that are aligned with a community’s needs, characteristics, and resources (8), and is considered a best practice for planning initiatives to improve community health (9).

## Methods

We conducted a primarily qualitative study that used 3 data sources: 1) focus groups with parents and youth, 2) interviews with community stakeholders, and 3) an intake survey of focus group participants.

We recruited study participants from 5 communities in Colorado with high rates of low-income households, physical inactivity, and child obesity. The sites we selected reflected the 4 geographic regions within the state: 2 communities were located in the same large city in the Urban Corridor, a small city was located in the Western Slope; and 2 small towns were located in each of the Mountain Range and Eastern Plains regions. Eligible parents were those who cared for a child aged 3 to 14 years, had an annual household income less than or equal to 200% of the federal poverty level, and spoke primarily English (or Spanish, in one Urban Corridor community). Youth were eligible if they were cared for by participating parents. A market research firm recruited families by calling residents whose names were in its proprietary database and advertising through social media and local organizations. For stakeholder interviews, we asked practitioners in the field to identify individuals involved with children’s physical activity in the study communities, which included staff members at recreation and health departments and nonprofit organizations.

In each community, we conducted 3 focus groups with parents (1 each with parents of children aged 3 to 7, 8 to 11, and 12 to 14); 1 focus group with 12- to 14-year-old youth from these families; and interviews with stakeholders. Researchers used semistructured interview guides to elicit discussion about barriers to and facilitators of physical activity and ways to increase children’s physical activity. The intake survey assessed participation in physical activities and perceived barriers to participation (6,10).

In total, we conducted 20 focus groups with 128 parents and 42 youth and interviews with 8 stakeholders from April to July 2016. Parents received $100 for participating and an additional $50 if their child participated. Participating youth and stakeholders received $50. We audio recorded and transcribed all focus groups and interviews. The Health Media Laboratory institutional review board approved all research protocols.

The 3 coauthors used thematic analysis to inductively analyze the qualitative data from focus groups and interviews. Applying the constant comparison method, we compared participants’ quotes and categorized them on the basis of their meaning (11). Next, we summarized findings for each group and community, reconciled discrepancies in meaning through discussion and systematic review, and merged findings across groups and communities under themes. We used SAS software version 9.4 (SAS Institute, Inc) to generate descriptive statistics for the survey data.

## Results

Most focus group parents were women (77%) (Table 1), half of families were white (54%), and a third of families were Hispanic (31%). Approximately 40% of the youth focus group participants were girls.

**Table 1:** Characteristics of Parents (N = 128) Participating in a Qualitative Study of Challenges and Opportunities for Promoting Children’s Physical Activity, Colorado, 2016^a^

Characteristic	Number of Families	Percentage of Families
**Total**	128	100.0
**Geographic location**		
Urban Corridor (English speaking)	25	19.5
Urban Corridor (Spanish speaking)	26	20.3
Mountain Range	15	11.7
Western Slope	29	22.7
Eastern Plains	33	25.8
**Age of child, y**		
3–7	46	35.9
8–11	40	31.3
12–14	42	32.8
**Sex of parent or caregiver**		
Female	98	76.6
Male	22	17.2
Not reported	8	6.3
**Race/ethnicity^b^**		
White	69	53.9
Hispanic or Latino	40	31.3
Black or African American	10	7.8
Asian	1	0.8
Other	1	0.8
Not reported	8	6.3
**Highest level of education**		
Less than high school	5	3.9
High school graduate	42	32.8
Some college	43	33.6
College graduate	30	23.4
Not reported	8	6.3
**Number of children in family**		
1	59	46.1
2	29	22.7
3	19	14.8
≥4	13	10.2
Not reported	8	6.3

a Eight parents who were screened onsite for eligibility in the focus groups did not provide data to the focus group recruitment staff, so their information, except for age of child, was not reported.

b We asked parents, “What is your ethnic background?” Parents could respond with more than one race or ethnicity with which they identified.

Parents had positive views toward physical activity; 81% reported that it is important that their child exercises regularly (data not shown). During focus groups, parents described their understanding of the value of regular physical activity, citing benefits to children’s physical health (maintaining a healthy weight and developing healthy habits), psychological well-being (improved mood and behavior), and social development (connecting with peers and developing social skills).

### Focus groups and interviews

We grouped themes according to family and community contexts, a process that is consistent with ecological models of health behavior (12). Although themes cut across levels, we identified 3 related primarily to the family context and 9 related primarily to the community context. We summarize themes, provide illustrative quotes, and present solutions identified from the focus groups in Table 2. The themes generally cut across age groups and communities; we note instances in which we observed variation across subgroups.

**Table 2:** Barrier Themes, Illustrative Quotations, and Solutions Identified From Focus Groups With Parents, Youth, and Stakeholders, Qualitative Study of Challenges and Opportunities for Promoting Children’s Physical Activity, Colorado, 2016

Barrier Themes/Family Context	Illustrative Quotations	Solutions
Parents’ work schedule	“I work full time, and me and my husband have one vehicle and we have 5 kids, and they’re all in sports. So we have to pick and choose who does what and when, and you know, to try to make it work, because there’s just no doing everything.” (Mother of 8- to 11-year-old)	Offer activities at times that accommodate working parents
		Encourage schools to host on-site activities after school
	“If I had a magic wand, I would say get some kind of bus that can go to all the different areas, pick up kids, take ’em where they want to go. . . . It breaks my heart when [my child’s] friends say . . . ‘My mom has to work so she can’t take us.’” (Mother of 12- to 14-year-old)	Provide subsidized transportation from school to activities
		Offer activities that serve children of multiple ages
Child’s lack of interest or apprehension	“One of our boys is bullied really bad at school, so he’s scared to play sports, because he doesn’t want to be on a team because of the kids that are bullying.” (Mother of 8- to 11-year-old)	Provide noncompetitive physical activity options that focus on fun and fitness
	“They’re so into their electronics, they want to sit at home and play the Xbox One and be on their tablet.” (Mother of 12- to 14-year-old)	
	“The group of kids that aren’t always into sports . . . those are the ones that get left out a little bit. [We need to] find alternative types of exercise and outdoor activities that they can do and that will help them get active.” (Community stakeholder)	
Youth’s school and family commitments	“I want to go to the park all the time, but I still have other priorities at home, like chores or homework or babysitting my younger siblings.” (12- to 14-year-old youth)	Create programs during school and out-of-school time that incorporate academics and exercise
**Community context**		
Lack of affordable options	“I think the city managers stand up and tell everybody, ‘Just because you can’t afford [the activity], doesn’t mean you can’t play it.’” (Father of 3- to 7-year-old)	Offer and publicize financial aid options that are accessible and noninvasive
	“I have to work to survive. It’s difficult to pay for activities and also take time to be there with her, and that’s why it seems hard for me.” (Mother of 3- to 7-year-old, Spanish speaking)	
	“My son this fall would like to do football now too, and just that is $90 and that doesn’t include the cleats that he’s going to need. And mouth guards. . . . I’m trying to figure out how I’m going to afford $90. Then my daughter, she wants to do volleyball . . . and it’s like, I don’t know how I’m going to, where the money’s going to come from?” (Mother of 8- to 11-year-old)	Create equipment exchanges
	“[I’d like] a trade-off or an equipment share or sports swap . . . because for growing kids it’s just impossible. You very rarely can use the same thing from year to year just because they’re growing.” (Father of 8- to 11-year-old)	
Traffic safety	“I think the town needs to consider sidewalks . . . [when] kids are walking after school or riding their bike, they’re going down the middle of the street. . . . Our boys are only allowed to play outside when we’re with them . . . it’s not safe [without sidewalks].” (Mother of 8- to 11-year-old)	Improve sidewalks and crosswalks
	“Where I live, it’s not very good to ride a bike. . . . I mean, if the sidewalks were a bit wider, perhaps. I think we also need those barriers so that the cars won’t drive by so fast. Because I am telling you, there’s been a few accidents involving kids riding their bikes.” (Mother of 12- to 14-year-old, Spanish speaking)	Implement traffic-calming measures
		Provide crossing guards near schools
Exposure to unsafe or illicit activity in public spaces	“They’re opening up [marijuana and alcohol shops] close to where the parks are. . . . They should be at a certain distance [from the parks].” (Father of 12- to 14-year-old)	Limit proximity of marijuana and liquor stores to parks and schools
	“[We] have a park across the street. . . . I prefer to go and walk with him there. . . . I don’t let him go by himself, I’m scared.” (Mother of 8- to 11-year-old, Spanish speaking)	Plan neighborhood watches to improve safety and security
		Improve lighting in public spaces
		Keep parks free of garbage, graffiti, and drug paraphernalia
		Provide clean and safe restrooms
		Consider increasing police presence
Limited access to high-quality facilities	“There’s rec centers [nearby], but I don’t want to put my girls in that situation, because they’re not the best rec centers. . . . So I have to look at other rec centers. Half the time, they’re full.” (Parent of 3- to 7-year-old)	Improve quality of existing indoor facilities
	“Sometimes at recreation centers that are a bit cheaper . . . you’re not so at ease because . . . the people that are teaching them let them do what they want and don’t pay attention to them.” (Mother of 3- to 7-year-old, Spanish speaking)	Hire staff who are qualified to work with children or improve training and supervision of staff
Transportation	“Transportation is a big thing, especially when you do have to go so far out of town ’cause we are in such a rural area. We have to get out of town to get our kids to play sports and play with other teams. That’s hard.” (Mother of 8- to 11-year-old)	Increase frequency and number of bus routes
	“I would love it if the schools coordinated with the rec centers and provided transportation. Wouldn’t it be great if from school, if there was a bus that went to the rec center?” (Father of 8- to 11-year-old)	Provide subsidized transportation to recreation facilities and after-school activities
Neighborhood inequities	“[It’s because] we’re of color; but you go down to the suburbs. . . . It’s kind of uncomfortable for the children to go down there. . . . It’s just that when you get out to certain areas, you’re not welcome… I told my daughter today, ‘Let’s go to one of the rec centers.’ She goes, ‘Nah.’ Just because . . . [she’s] not comfortable.” (Father of 8- to 11-year old)	Create and improve the quality of indoor and outdoor facilities in lower-income neighborhoods
	“We are campaigning to have a rec center in [a low-income] neighborhood. Despite having the most kids and the most child obesity, we do not have a community rec center. We’re one of the least served, so we are . . . hoping to have it on the next bond so that we get a rec center.” (Community stakeholder)	
Limited program availability	“There’s this small window of time on a certain day [when you can sign up for recreation center programs], and it’s always during the middle of the day. . . . And all of the times that are convenient for busy families are full within a half hour.” (Mother of 3- to 7-year-old)	Offer more activities for young children
	“It’s hard in the summer to get them where they need to be — all the things you want them to do so that they’re active and busy. And you know? That’s really stressful to me in the summertime. I’m working and he’s not doing anything. So again, one of those rec centers would be really nice.” (Father of 12- to 14-year-old)	Expand use of existing facilities (schools, churches) to offer activities in winter
	“Nearby my house there are 3 churches that are huge. . . . I ask myself, ‘That building is empty. . . . Why don’t they open it up so we could come and play, especially in the winter?’” (Mother of 12- to 14-year-old, Spanish speaking)	Build partnerships with community organizations such as public libraries to promote physical activity in summer
Lack of information	“I mean, there are options, but what I [am saying] is that you also have to make an effort and look for the places. . . . The school doesn’t announce the rec centers.” (Mother of 8- to 11-year-old)	Create user-friendly, up-to-date electronic listings with current physical activity offerings and locations
	“The city has a website and it has a calendar [of events] . . . but it’s really slow. It’s not user-friendly and I wish that they could have a kid’s corner. . . . They could pull . . . all the information together for children in one spot. . . . [Now, the site] seems geared toward the retirement community.” (Mother of 3- to 7-year-old)	Publicize opportunities in local newspapers
	“I think another thing [that would help me] is advertising or sending out information to get people to go. . . . I’ve lived here 2 years. I know where the rec center is, but I don’t know what they offer.” (Mother of 12- to 14-year-old)	Support social media networks in which parents can share information with each other
Limited engagement with community	“[Local planners] need to tailor to the needs of each community because something that works one place might not work somewhere else.”	Solicit community input such as conducting “listening campaigns” when planning activities

### Family context

#### Parents’ work schedule

Several parents indicated that their work schedules make it challenging to enroll children in organized activities or to be active with children at home. Many activities take place after school, which is not feasible for parents whose work schedule precludes them from transporting children to activities in the afternoon. Parents who work at night or on the weekends, which was common among parents in a rural Mountain Range community, have less time to be physically active with their children. Parents’ and stakeholders’ solutions for addressing scheduling constraints included offering more activities in the evening or during the weekend, offering transportation from school to recreational facilities, partnering with schools to expand offerings in school facilities during out-of-school time, and developing activities that serve children of multiple ages.

#### Child’s lack of interest or apprehension

Parents cited children’s lack of interest in available activities, preoccupation with electronic devices, and fear of being bullied as reasons they do not participate in physical activities. As solutions, parents, youth, and stakeholders recommended increasing activities that focus on fun and fitness, such as dance classes, or sports teams that emphasize social–emotional aspects rather than competition.

#### Youth’s school and family commitments

Youth described avoiding activities because they conflict with the time when they complete homework. Others said family obligations, such as caring for siblings or chores, limit their participation. To address these conflicts, parents suggested offering activities during non–after-school times that enable children to participate in academic work and physical activity.

### Community context

#### Lack of affordable options

Parents in all communities and across age groups cited the high costs of enrolling in activities, purchasing equipment, and membership fees as barriers to children’s participation in physical activity. In the Urban Corridor and Mountain Range communities, parents reported that this barrier was exacerbated by the high cost of living in their communities. Some communities offer financial assistance to families, but parents and community stakeholders described the application as a complicated process that required families to divulge private information. As potential solutions, parents and stakeholders suggested that communities make these activities more affordable, for example, by offering low-cost or free activities sponsored by towns or community organizations, creating equipment exchanges, and offering financial aid that is accessible and noninvasive.

#### Traffic safety

Parents commonly cited safety hazards related to cars and traffic — particularly in the Western Slope and Urban Corridor communities — as barriers to their allowing children to play or travel outside. In particular, parents identified the absence or poor condition of sidewalks and crosswalks as a barrier to playing outside and biking and walking to parks. For example, one parent said, “The streets aren’t very safe. Cars drive by too fast. . . . Every day as soon as he comes home, [my son would like] to go out on his bike but it’s not very safe.” Parents suggested that sidewalk improvements, crosswalks, traffic-calming measures, and crossing guards near schools would ease traffic-related concerns.

#### Exposure to unsafe or illicit activity in public spaces

Concerns about neighborhood safety prevent parents from allowing children to play in parks and playgrounds, even with a supervising adult. Communities in all 4 regions reported this concern. Parents and youth expressed concerns about adults or older teens they perceived as threatening, peer violence, unleashed dogs, and poorly maintained public spaces and equipment. In the Urban Corridor community, parents expressed concern with drug use near parks, especially now that recreational use of marijuana is legal in Colorado. One stakeholder corroborated this concern, saying that in his community “parks have become the place where negative activity happens.”

Parents’ ideas for improving safety in outdoor spaces included organizing neighborhood watches to promote trust among neighbors and limiting the proximity of marijuana and liquor stores to parks. Parents had mixed opinions about the value of increasing police presence in their neighborhoods. Parents and stakeholders suggested making sure that public spaces are free of garbage, graffiti, and drug paraphernalia; improving lighting; and providing clean and safe restrooms. Stakeholders also proposed stationing child care professionals at parks to supervise and facilitate play.

#### Limited access to high-quality facilities

Across all communities, parents mentioned a lack of indoor recreational facilities as a barrier. Such facilities provide space for activity during colder months, host organized activities, and serve as a hub for families to socialize. Some parents in the Urban Corridor communities are reluctant to use local recreation centers, because they are poorly maintained or perceived to be unsafe because of surrounding neighborhoods. One mother described how illicit activity in the neighborhood, such as drug dealing or peer violence, spreads into the recreation center, commenting, “I don’t want to put my girls in that situation.” Parents in these communities also had concerns that facility staff do not have sufficient skills for working with children.

Parents in all communities expressed a need for more high-quality indoor facilities and improvements to existing facilities, including maintenance and modernization. They also recommended that centers hire better-qualified staff or improve training and supervision.

#### Transportation

Parents reported the need to travel to access physical activity programs or high-quality facilities; this was a barrier reported in all communities. Transportation-related challenges included time spent driving or riding public transportation and fuel and bus pass costs. Parents in the Urban Corridor communities said they travel to other sections of the city or suburban communities, whereas those in the Mountain Range and Eastern Plains rural communities travel longer distances to adjacent towns or states.

Parents recommended offering safe and subsidized options, such as school district–sponsored buses, for transporting children to activities after school. Community stakeholders and parents said increasing the frequency and number of bus routes and offering lower-cost transportation would improve children’s access to physical activity opportunities.

#### Neighborhood inequities

Parents and community stakeholders in the 2 Urban Corridor communities noted inequities in the quality of recreation centers and outdoor spaces in their communities relative to other neighborhoods. One parent said, “All the parks that are being built are in areas where the people have a higher income; I don’t know why this is, but that’s how it is.” Parents said their facilities were poorer-quality, and they had travel to other parts of the city to access higher-quality parks and playgrounds. One parent said that, as people of color, his family feels unwelcome in recreation centers in higher-income neighborhoods. Proposed solutions centered on improving parks and recreation centers so that children in these neighborhoods had the same opportunities as children in higher-income neighborhoods.

#### Limited program availability

Parents reported that there are limited program options during specific times of year (winter and summer) and for certain populations (preschool-age children). Registration often occurs during the workday, and programs fill up quickly. Parents in the Urban Corridor communities focused on the lack of summer offerings. To address the need for summer programming, one stakeholder highlighted a partnership with the local library that orients youth to new sports and offers supplies. To increase activities during the colder months, parents and stakeholders in the Western Slope community recommended using existing facilities such as schools or churches for indoor play spaces. For preschool-aged youth, parents recommended designing facilities for young children or offering dedicated preschool hours in existing facilities.

#### Lack of information

Parents reported challenges in finding complete information about opportunities for children’s physical activity. During the focus groups, parents said that there is no central repository for learning about children’s activities and that they have to rely on word of mouth. Parents suggested maintaining up-to-date electronic resources with listings of physical activity programs and publicizing opportunities in local newspapers and guides. Parents also recommended improving families’ abilities to communicate with one another through social media (eg, Facebook groups, email Listservs).

#### Limited engagement with community

Both parents and stakeholders across all communities indicated that program planners often design activities without input from parents and that this leads to underutilization of activities or facilities. One mother in the Urban Corridor community said she wished “that Parks and Recreation would take more into consideration the needs of the community, because many times . . . they don’t.” Community stakeholders indicated they had success when community leaders held meetings or “listening campaigns” with parents, youth, and other users of planned programs or facilities. Parents and stakeholders alike discussed that when this did not happen, investments fell short of their intended goals (eg, families underutilizing community activities).

### Parent survey

The parent survey results demonstrate the frequency with which parents believe that 22 prespecified factors were challenges to children’s physical activity (Table 3). The most commonly cited barriers — that 60% or more of parents agreed limit their child’s physical activity — were cost, including enrollment fees and sports equipment; safety, including drivers not looking out for children and driving too fast; and access to indoor facilities near home. The least commonly cited barriers — that 20% or fewer cited as a barrier to physical activity — were access to parks and playgrounds to which children can walk or bike and lack of sidewalks.

**Table 3:** Parent Survey Results (N = 126) on Perceived Barriers to Physical Activity, Qualitative Study of Challenges and Opportunities for Promoting Children’s Physical Activity, Colorado, 2016

Barrier	% of Parents Who Strongly Agree or Agree That Item Is a Barrier to Their Child’s Physical Activity	Ranking of Frequency
**Cost**		
I cannot afford enrollment fees for after-school programs/camps.	68.3	2
I cannot afford enrollment fees for sports and clubs.	67.5	3
I cannot afford equipment and gear for sports teams.	62.7	4
I cannot afford activity-related equipment such as bicycles.	43.7	7
**Safety**		
Drivers don’t look out for children playing.	73.0	1
Cars drive too fast for my child to play near the road.	61.1	5^a^
There is too much traffic for my child to play outside.	33.3	14
It is unsafe for my child to play outside.	30.2	16
I worry that my child will get injured during sports and physical activities.	21.4	20
**Access to parks and facilities**		
There are few indoor facilities near my home.	61.1	6^a^
I have no backyard for my child to play in.	31.0	15
There are no sidewalks for my child to walk or bike on.	18.3	21
There are no parks or playgrounds that my child can walk or bike to.	12.7	22
**Availability of programs**		
Hours for after-school/summer programs are not flexible.	39.7	11
There aren’t many teams/programs in our neighborhood.	38.1	12
There are no teams/clubs for activities my child likes to do.	23.8	18
**Parent schedules**		
I work and have little time at the end of the day.	40.5	9^b^
It is difficult to coordinate activities for children of different ages.	40.5	10^b^
I have no energy to help my child be active.	22.2	19
**Information**		
There isn't much information on sports/activities available.	42.9	8
I don’t know how to get my kids to be active in winter.	27.0	17
**Other**		
There are no children with similar interests in our neighborhood.	34.1	13

a,b Two sets of barriers were tied in their ranking; these ties are denoted by footnotes a and b.

## Discussion

This formative study of parents and children provides insight into the challenges low-income families in Colorado face in supporting children’s physical activity and describes their recommendations for addressing these barriers. It confirms several barriers already documented in the literature, such as neighborhood safety, program cost, and access to facilities (4,5). It also identifies less frequently documented factors, such as the difficulty obtaining financial aid and the lack of centralized information. Most of these barriers are at the community level rather than the family level, and nearly all solutions are at the community level. Although our study identified a common set of barriers across the Colorado regions, we also identified barriers that were most salient to families living in specific regions. For example, traffic safety was most frequently cited in the Urban Corridor communities, whereas distance to activities was cited in the rural Eastern Plains and Mountain Range communities.

One key finding is that this sample of parents recognized the importance of physical activity. Many physical activity interventions focus on individual-level factors, such as counseling families about the benefits of physical activity (13), but most parents indicated they believe it is important that their child exercises regularly. Despite this knowledge, their children are not as active as the parents would like, and this may be caused by barriers in their surrounding community, with 9 of the 12 barriers that parents identified being at the community level. This finding suggests that community-level interventions that address the affordability, accessibility, and safety of physical activity options may be more successful than those that target children’s or parents’ knowledge.

A strength of this study is that we collected information on parents’ views on barriers to physical activity through focus group discussions and a parent survey. The survey results reflect the magnitude of the concerns raised during the focus groups. Two prominent challenges raised during the focus groups — cost of activities and traffic safety — were the most frequently cited barriers in the parent survey, with more than 60% of parents endorsing 5 items related to these types of challenges. In contrast, 2 prominent barriers raised during the focus groups — having parks or playgrounds that are accessible by walking or bicycling and having sidewalks — were the least frequently cited barriers in the parent survey. It is unclear why these factors emerged as barriers during the focus groups and were not cited as frequently in the survey, but it is worth noting that the survey items address the proximity and presence of public spaces and sidewalks and not necessarily the quality and maintenance of this infrastructure. Our findings are consistent with those of previous research that suggest that in developing interventions to promote physical activity, addressing both proximity and quality is important (14,15).

This study also has limitations. We collected data from a small sample of families residing in 5 communities, and the groups may not be representative of the barriers faced by other families in Colorado or other states. Nevertheless, this project sampled participants in urban and rural locations, and our study findings mirror other findings of other studies about parents’ concerns with children’s safety because of traffic and financial barriers to participation (4–7).

A basic premise for this formative study of Colorado families is that community engagement and stakeholder input are essential for planning initiatives to improve community health. Parents and stakeholders validated this premise during the focus groups and interviews, emphasizing the importance of ensuring that specific investments are community-driven and describing instances when programs went underused because they did not incorporate community input. Both the methods used and findings of this study underscore the importance of funders and public health planners soliciting input from families and stakeholders when undertaking large-scale programs and initiatives to ensure that these plans meet the needs of their target population.
